# Congruent Head Orientation in the Absence of Sight Facilitates Motor Performance

**DOI:** 10.3390/vision10040062

**Published:** 2026-09-01

**Authors:** Jennifer Stevens, Emma Sare Stephenson, Cory Winkler, Ipsita Bhakre, Mia Lawrence

**Affiliations:** 1 Department of Psychological Sciences, William & Mary, Williamsburg, VA 23187, USA; 2 Department of Psychology, University of Virginia, Charlottesville, VA 22903, USA; 3 Department of Health Sciences and Research, Medical University of South Carolina, Charleston, SC 29425, USA

**Keywords:** head orientation, blindfolded, visual simulation, eye-hand coordination

## Abstract

(1) Background: During fine motor object manipulation, head orientation towards the workspace allows the small receptive fields in our cone-rich fovea to perceive details required to complete the task. Here, we investigated whether a head orientation benefit remains even when sight is unavailable. (2) Methods: Blindfolded, sighted participants reconstructed three-dimensional objects in 45-degree leftward, centered (0-degree), and 45-degree rightward workspaces in conditions when their head was oriented either towards (congruent) or away from (incongruent) the workspace. (3) Results: Performance times were found to be significantly faster when the head was oriented towards the workspace even though vision was occluded. Moreover, the same benefit was found regardless of workspace location, suggesting the effect is unrelated to comfort or experience. (4) Conclusions: Our findings demonstrate a motor performance time benefit when there is a congruency between head orientation and workspace location in the absence of sight. Head orientation may align the visual system with the target of action, thereby enabling representational detail akin to that provided by the small receptive fields in the cone-rich fovea. This is the first study demonstrating the benefit of head orientation in blindfolded, sighted individuals.

## 1. Introduction

Eye–hand coordination is a central tenet of successful motor control. Neural pathways linking high-acuity visual information with sensorimotor systems enable the remarkable precision of human manual dexterity. High-resolution visual encoding depends on the small receptive fields found in the cone-rich fovea [1,2]. In the central fovea, signaling from cone photoreceptors to retinal ganglion cells approaches a one-to-one relationship, preserving spatial detail as visual information enters the optic nerve [2,3]. Tasks requiring fine spatial precision, such as threading a needle, rely on this kind of detailed encoding to achieve accuracy. In contrast, much of the peripheral visual field is represented by neurons with larger receptive fields, resulting in lower spatial resolution [4]. Although fine detail is not explicitly encoded across the periphery, perceptual completion and predictive processing allow observers to experience the visual world as a unified whole [5,6,7].

Perception and action are tightly coupled through recurrent feedforward and feedback pathways that support continuous sensorimotor integration [8]. Because neural transmission and sensory processing introduce processing delays, the nervous system relies on internal forward models to predict the consequences of movement before complete feedback becomes available [8,9]. These predictive processes share neural mechanisms within action planning and motor imagery, just as visual imagery and perception have overlapping substrates and adhere to the same rules of action [10,11]. These real and imagined processes operate similarly across both spatial and temporal domains. Spatially, the brain constructs internal representations of the environment that guide movement and can be generated from perception, memory, or imagery. Temporally, predicted sensory consequences are continuously compared with incoming sensory feedback, allowing motor commands to be refined online and enabling smooth, adaptive interactions with the environment. The demands of baseball batting illustrate the speed of this process: a 95 mph (153 km/h) pitch reaches the home plate in approximately 400 ms, leaving little time for pure reactive control. Instead, batters rely on predictive processing to initiate the swing while using ongoing visual information to make rapid adjustments as the ball approaches [12,13].

Alongside the perception–action framework, the literature on embodied cognition identifies spatial cognition, action planning, and memory as being shaped by the position and movement of the body, with posture and orientation contributing to how space is encoded and utilized [14,15]. The head, eyes, and body form an integrated sensorimotor system in which orientation both reflects and constrains perception and action. Experimental studies have shown that head orientation influences auditory localization and spatial judgments by altering the body-centered reference frame used for target localization [16,17]. Similarly, eye, head, and hand movements are tightly coordinated during visually guided reaching and manipulation, with head stabilization and orientation contributing to movement accuracy and efficient online control [18,19].

In most situations, eye gaze and head orientation precede and predict subsequent actions, reflecting the anticipatory nature of sensorimotor planning rather than merely responding to environmental events [20]. On this view, head orientation is not simply a consequence of visual attention but an integral component of embodied sensorimotor control. If head orientation contributes to maintaining body-centered spatial representations during action, its functional role should persist even when vision is absent. Research in visually impaired contexts provides an opportunity to examine this possibility by dissociating the orienting of the head from the acquisition of visual information.

Relatively few studies have examined head orientation during manual task performance in blind individuals, but the available evidence suggests that head orientation serves a functional role beyond visual acquisition. Observational work by Wolk and colleagues [21] found that congenitally blind individuals spontaneously oriented their heads toward a manual workspace while performing a bead-stringing task, despite receiving no visual information and often without awareness of doing so. The authors proposed that head orientation may contribute to embodied spatial attention or the maintenance of body-centered spatial representations during manual action. This finding was published as a conference abstract only but is one of the few studies that directly observed spontaneous head orientation during manual tasks in congenitally blind individuals. Other experimental studies indicate that head orientation influences nonvisual spatial processing. Manipulating head orientation in sighted participants systematically affects performance on a tactile localization task [22]. Head orientation affected performance during a raised line-drawing identification task in early-blind, late-blind, and sighted participants. Although developmental visual experience affected the spatial reference frames used during haptic perception, the benefit of facing the object was evident even in the absence of vision, suggesting that head orientation contributes to embodied sensorimotor processing beyond its role in visual guidance [23]. Research on auditory localization further demonstrates that head orientation contributes to spatial coding in blind individuals. For example, Lewald [15] showed that changes in head position systematically alter sound localization, indicating that head orientation forms part of the body-centered reference frame used to represent auditory space.

Taken together, these findings suggest that head orientation remains an integral component of sensorimotor organization even when vision is absent, supporting the possibility that orienting the head toward a workspace facilitates internal spatial representations during manual performance. The role of previous visual experience appears to have an influence on head orienting strategies. Individuals who become blind later in life often retain visually acquired patterns of orienting during social interaction and object-directed behavior, whereas congenitally blind individuals rely more heavily on proprioceptive and auditory information to calibrate spatial representations [16,24]. Nevertheless, both groups demonstrate robust body-centered representations of peripersonal space, indicating that accurate spatial cognition does not depend exclusively on visual experience [25].

Although relatively little research has examined head orientation toward a manual workspace directly, the available evidence suggests that orienting the head toward the locus of action serves functions beyond visual acquisition. Instead, head orientation appears to contribute to the maintenance of spatial reference frames and the predictive control of movement through embodied sensorimotor mechanisms. This perspective motivates the present study, which directly examines whether head orientation without visual information affects motor performance. We hypothesized that performance times would be fastest in congruent conditions compared to incongruent conditions in eyes-closed conditions, while it is expected that eyes-open conditions will yield faster performance times than eyes-closed conditions overall.

## 2. Materials and Methods

### 2.1. Participants

Participants were recruited via an online sign-up system (Sona systems, www.sona-systems.com accessed on 20 July 2026) associated with a college Introduction to Psychology course sequence. Participants received 0.5 research engagement credits in exchange for their participation in this study. Participation was voluntary. Prior to study start, participants reviewed a summary of the method and gave their informed consent. The sample consisted of 24 healthy, college-aged participants (10 Male, 14 Female), 12.5% confirmed as left-hand dominant. The study was approved by the William & Mary IRB.

### 2.2. Materials and Apparatus

**Figure 1:**
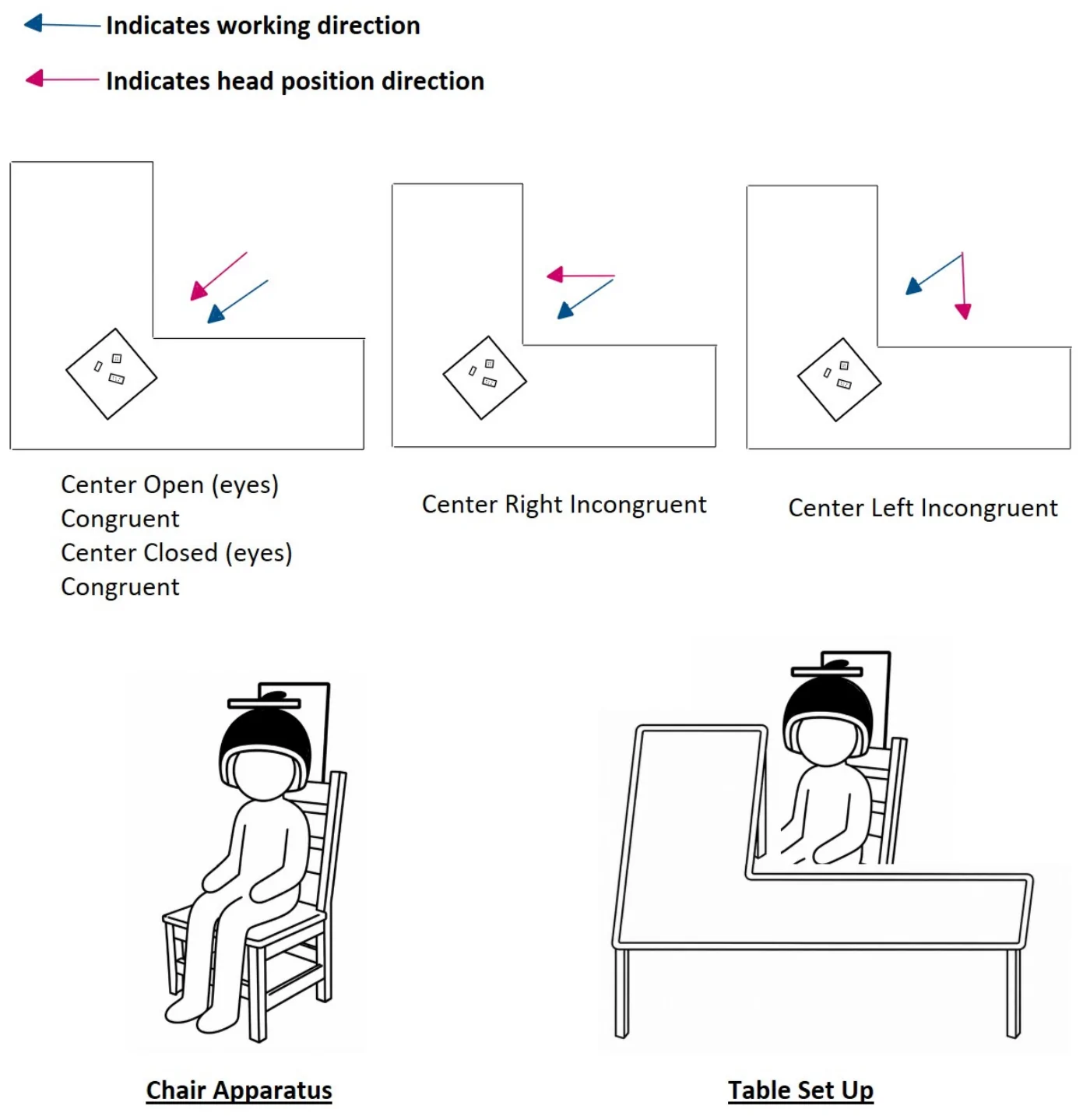
The experimental set-up using center head orientation as an example. Participants were seated in a chair with a helmet affixed to its base (**lower left**). The chair and the participant’s body position remained in the same position for the duration of the experiment (**lower right**). Along the top: overhead views of the congruent and incongruent trials, with both head and workplace location indicated with red and blue arrows.

A custom-built head orientation apparatus was used to control placement during each of the trials. A solid wooden chair served as the base. A thick metal dowel that could be raised and lowered based on participant height supported a metal rotator from which a motorcycle helmet was suspended. The metal rotator could be moved 45 degrees left and 45 degrees right from center by raising a lock bar. After the lock bar was lowered, the helmet could not be moved. Participants placed a shower cap over their hair and a blindfold around their eyes before placing their heads in the helmet. Once a participant was in the helmet securely, their head was moved to the placement for the first group of trials (left, center, or right), depending on trial order. In between trials, and during eyes-open trial points, participants lowered the eye mask to their chin. The head orientation apparatus faced a corner desk, allowing symmetrical access to left, center, and right workspace areas identified by a 12″ × 12″ cork mat. Figure 1 displays a schematic of the apparatus and each of the trial workspace conditions using the center head orientation as an example. The chair remained in the same position throughout the experiment. That is, the participant’s body remained in line with the chair and toward the center of the set-up, while head orientation and workspace location were manipulated independently between trials.

**Figure 2:**
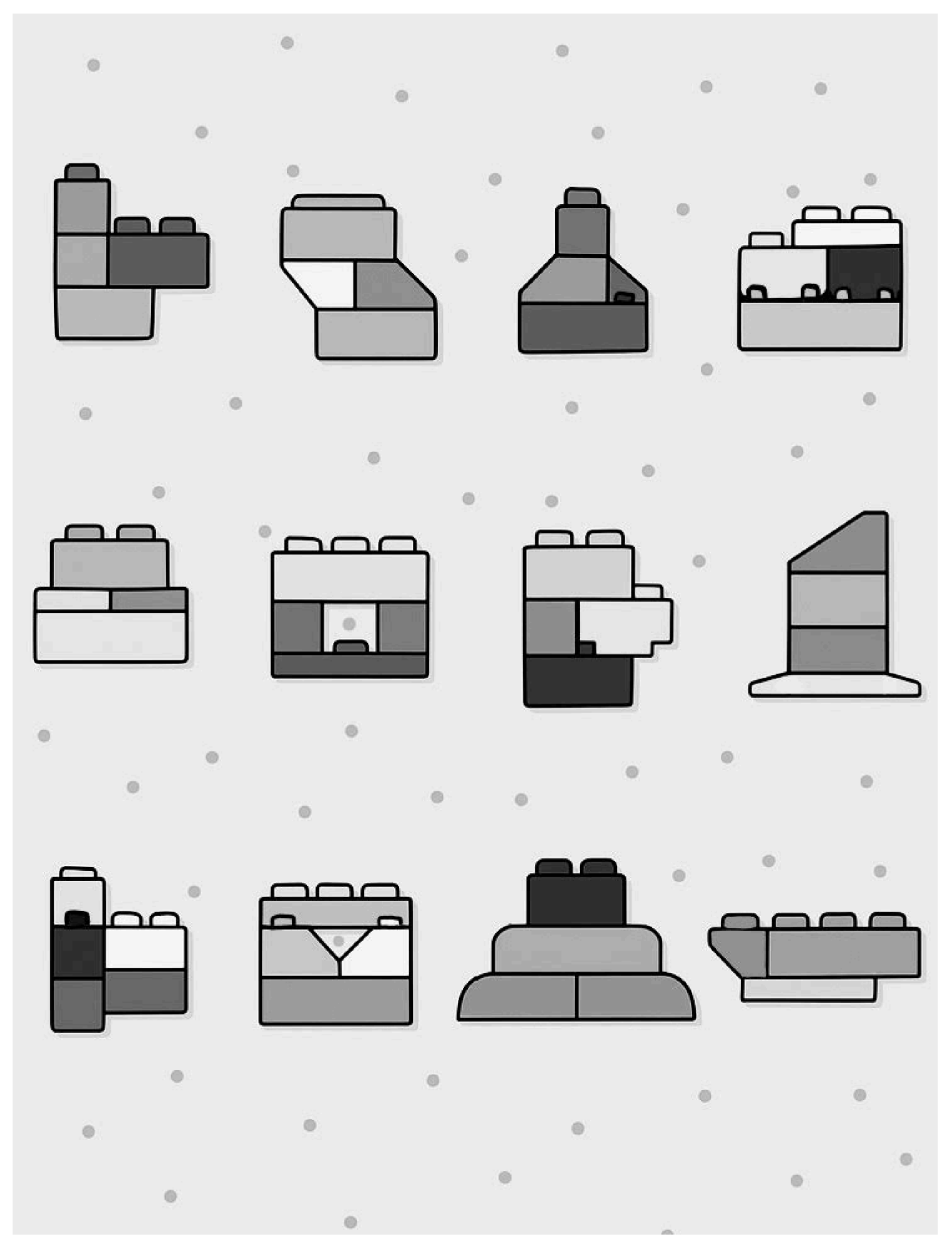
Black and white rendering of the twelve 4-piece Lego constructions used as stimuli. Participants examined the structure with eyes open in trial position before covering their eyes with the blindfold and receiving the four separate pieces to reconfigure into the same construction.

Traditional Lego pieces were used for the object reconstruction task. Figure 2 displays the twelve Lego constructions used. The same twelve constructions were used and presented in the same order for each participant regardless of head orientation or congruency trial type. A SONY camcorder was used to video record the experimental sessions (Sony Corporation, Tokyo, Japan).

### 2.3. Design and Procedure

Three head orientations—left (L), center (C), and right (R)—were tested. The order of head orientations was randomized across participants, yielding six possible sequences (LCR, LRC, CLR, CRL, RLC, and RCL). Each head orientation included four trials: two congruent trials, in which head orientation and workspace location matched (e.g., head oriented to the center with the workspace positioned at the center), and two incongruent trials, in which head orientation and workspace location differed (e.g., head oriented to the center with the workspace positioned to the left or right). Congruent trials were completed once with eyes open and once with eyes closed, whereas both incongruent trials were completed with the workspace positioned in one of the two nonmatching locations. Trial order within each head-orientation condition was randomized. After completing the four trials for one head orientation, the experimenter repositioned the participant’s head to the next orientation until all three head orientations had been tested, for a total of 12 trials.

In each trial, participants visually inspected a four-piece LEGO construction and were then asked to reconstruct it from memory. During the inspection phase, the completed LEGO model was placed on a cork workspace mat positioned in the direction of the participant’s head orientation. For example, in a left-orientation trial, the participant’s head was turned to the left, and the workspace was positioned to the left. Participants explored the model with both hands while visually inspecting it for 15 s and were instructed to learn the configuration well enough to reproduce it during the reconstruction phase.

Following the inspection period, the eye mask was adjusted according to the trial condition so that participants either retained vision (eyes-open trials) or were fully occluded (eyes-closed trials). The experimenter then repositioned the workspace to the location specified by the trial’s congruency condition and placed four unattached LEGO pieces, identical to those used in the model, onto the center of the workspace by overturning a small cup. After the pieces were deposited in a random arrangement, participants were instructed to reach forward and reconstruct the model from memory. Upon completion, they placed the reconstructed model on the workspace mat. The entire experimental session was video recorded to permit offline coding of reconstruction times for each trial.

## 3. Results

### 3.1. Analysis Procedure

Each video was re-recorded with a timer that presented milliseconds. Two independent coding teams analyzed the new recordings using Windows Media Player 12, which allowed frame-by-frame analysis. Participants’ first and final touches were coded and entered using a second/millisecond format (e.g., 570.12). Inter-rater reliability was calculated using the intraclass correlation coefficient (ICC) calculation found in IBM SPSS (Version 26), with a two-way mixed model and single measures. The ICC (2,1) was 1.0, with a 95% confidence interval of 0.60 to 0.85, indicating excellent rating reliability. Average start and end times were calculated for each trial based on the coded values entered by Rater 1 and Rater 2 and entered in Excel and SPSS for statistical analysis.

### 3.2. Experimental Results

Performance times across the three eyes-open congruent conditions did not differ significantly across the three head orientations, *F*(2,22) = 2.38, *p* = 0.116. Participants worked at similar speeds in the left (M = 12.53, SD = 8.7), center (M = 11.80, SD = 5.84), and right (M = 9.79, SD = 2.80) orientations when their eyes were open, with a trend towards faster movements as the workspace shifted from left to right. Similarly, there was no difference in performance times in the eyes-closed congruent condition across the three head orientations, *F*(2,22) = 0.342, *p* = 0.714; left (M = 25.55, SD = 18.36), center (M = 22.57, SD = 8.30), right (M = 25.08, SD = 14.04). There was also no main effect for the eyes-closed incongruent conditions, *F*(2,22) = 1.594, *p* = 0.226; left (M = 27.56, SD = 12.16), center (M = 26.90, SD = 12.58), right (M = 32.54, SD = 19.55). The eyes-closed incongruent mean collapses across two incongruent eyes-closed conditions for each head orientation (e.g., the left eyes-closed incongruent mean averages across the center and right eyes-closed incongruent trials). The lack of a consistent pattern in performance times across these three contexts (eyes-open congruent, eyes-closed congruent, and eyes-closed incongruent) suggests that the central position provides no benefit (i.e., there is no effect of comfort).

There was a significant difference in overall performance times between the eyes-open and eyes-closed congruent trials, *t*(23) = −6.932, *p* < 0.001. Participants performed twice as fast in the eyes-open condition (M = 11.38, SD = 3.85) as in the eyes-closed condition (M = 24.62, SD = 11.07). The same effect held in all three orientations: left, *t*(23) = −3.44, *p* = 0.002; center, *t*(23) = −6.78, *p* < 0.001; right, *t*(23) = −5.44, *p* < 0.001. There was also a significant difference in overall performance times between the eyes-closed congruent and eyes-closed incongruent trials, *t*(23) = −2.173, *p* = 0.04. When considering each orientation separately, there was no difference in performance times between the eyes-closed congruent and eyes-closed incongruent trials at left, *t*(23) = −0.567, *p* = 0.577. In the center orientation, there was a significant difference in performance times between the eyes-closed congruent and eyes-closed incongruent trials, *t*(23) = −2.250, *p* = 0.02. And, in the right orientation, the difference between the eyes-closed congruent and eyes-closed incongruent trials approached significance, *t*(23) = −1.899, *p* = 0.07.

In line with our hypothesis, there was a main effect of congruency: *F*(1,22) = 5.816, *p* = 0.025. Participants worked significantly faster in the eyes-closed congruent conditions (M = 24.62, SD = 11.07) compared to the eyes-closed incongruent conditions (M = 29.15, SD = 12.96). In the absence of sight, motor performance time benefits when the head is oriented toward the workspace. In the left and right orientations, there was the opportunity to compare performance times in first- and second-order derivations. One might expect performance times to increase as head orientation moves away from the workspace. However, neither comparison revealed a significant difference: left, *t*(23) = 0.352, *p* = 0.72 and right, *t*(23) = −1.532, *p* = 0.139. Increasing the angular deviation to 90° did not produce additional slowing when compared to the 45° deviations. A Bonferroni correction may be applied to each family of three follow-up t-tests to account for the increased familywise Type I error possibility. Accordingly, the conventional α = 0.05 criterion is divided by three, yielding a Bonferroni-adjusted significance threshold of α = 0.0167. While none of the follow-up t-tests meet this significance level, we are confident in the validity of our results. The pattern of performance times across the three head orientations is consistent: there is an increase in the incongruent conditions, suggesting that a Type I error is not likely to be present.

**Figure 3:**
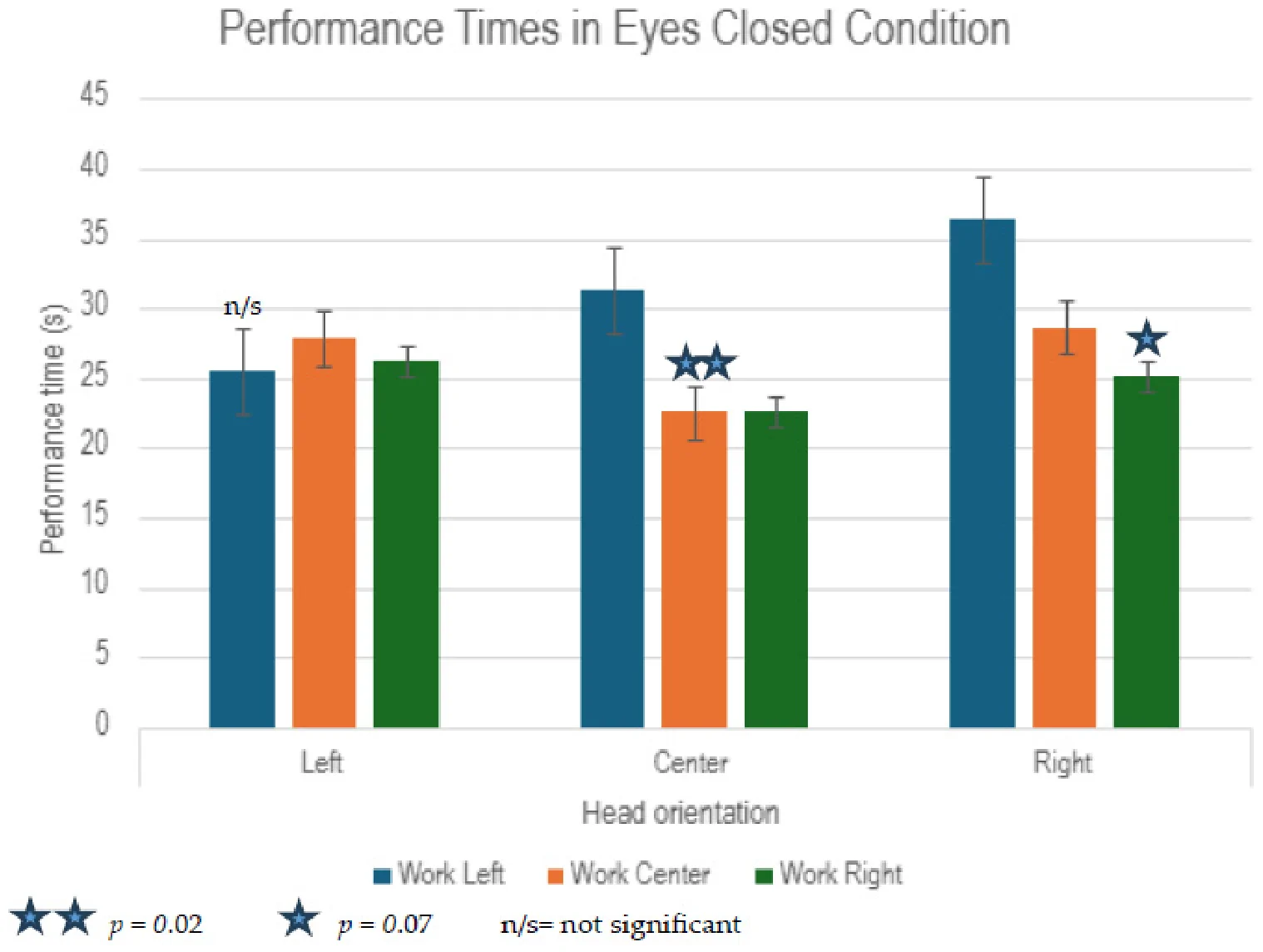
Mean performance times (seconds) for the three head orientations (horizontal axis identifying left, center, and right head orientations) across the three eyes-closed workspace conditions (bars representing Work Left, Work Center, Work Right). Significance between congruent and incongruent trials for the center and right orientations is indicated by the icon above the congruent trials for each head orientation.

Figure 3 depicts the overall performance times across the three orientations and congruency conditions. In the left orientation, participants worked fastest in the congruent workspace condition and slowest in the center workspace condition (M = 27.88, SD = 12.04). In the center orientation, the leftward workspace yielded a much slower performance time (M = 31.32, SD = 19.06), while the rightward workspace (M = 22.59, SD = 9.39) yielded a nearly identical performance time to the congruent condition (M = 22.57, SD = 8.30). In the right orientation, performance times slowed in the center workspace condition (M = 28.67, SD = 13.12), and the decrement was over twice as large in the left workspace condition (M = 36.41, SD = 30.0). Only the right orientation resulted in progressively longer performance times as eyes-closed and head–workspace angular deviation increased from 0° to 45° to 90°.

## 4. Discussion

The present study examined whether head orientation influences manual task performance when visual information is unavailable. Consistent with the primary hypothesis, participants reconstructed 4-piece models significantly faster when head orientation was congruent with the workspace location as compared to when head orientation and workspace location were incongruent. Importantly, this effect was observed while participants were blindfolded during task execution, indicating that the benefit of orienting the head toward the workspace cannot be attributed to the acquisition of visual information. The findings suggest that head orientation significantly contributes to the organization of sensorimotor behavior even in the absence of vision.

As expected, participants completed the task substantially faster when vision was available during reconstruction than when blindfolded. This finding is consistent with extensive evidence demonstrating the critical role of visual feedback in online motor control [8,25]. Visual information permits continuous correction of movement trajectories and object manipulation, resulting in more efficient task performance. The approximately twofold increase in reconstruction time under blindfolded conditions reflects a reliance on proprioceptive, tactile, and memory-based processes when visual feedback is unavailable. Our visual system provides maximum detail in the center of our gaze, and this directly enhances our ability to perform precise movements with our hands. That head orientation facilitates motor coordination under blindfolded conditions suggests that this orientation benefit extends to contexts during which eye–hand coordination is visually simulated.

The primary contribution of the present study lies in the comparison of congruent and incongruent head orientations under eyes-closed conditions. Participants consistently required less time to complete the reconstruction task when the head remained oriented toward the workspace than when the workspace was relocated outside the participant’s line of regard. Because visual input was eliminated during task performance, the congruency effect cannot be explained by differences in visual acuity or gaze-dependent visual processing. Instead, the results are consistent with theories of embodied cognition, which propose that cognitive processes are grounded in the body’s interactions with the environment [14,15]. Within this framework, head orientation may serve as part of an integrated body-centered reference frame that supports spatial memory and action planning even when vision is absent.

The findings also align with models of predictive sensorimotor control. Contemporary theories propose that movement depends on internal forward models that continuously predict the sensory consequences of action while integrating proprioceptive, vestibular, tactile, and visual feedback [8,9]. Although the present experiment did not directly measure predictive processes, orienting the head toward the workspace may facilitate the maintenance of these internal spatial representations, thereby reducing the computational demands associated with transforming information between head-centered and body-centered reference frames. On this view, head orientation functions not simply as a mechanism for acquiring visual information but as an embodied component of the sensorimotor system that contributes to efficient encoding of mentally simulated visual perceptions, and subsequent movement planning and execution.

An alternative explanation is that congruent head orientation reduces biomechanical demands associated with reaching or manipulating objects. However, this interpretation appears unlikely to account fully for the findings. Performance did not differ across the left, center, and right head orientations in either the eyes-open or eyes-closed congruent conditions, indicating that no particular head position, including the centrally aligned position, introduced an advantage. If biomechanical comfort or postural stability were the primary determinant of performance, faster completion times would be expected in the center orientation, where neck rotation is minimal. Instead, performance depended on the relationship between head orientation and workspace location rather than on head position itself.

Inspection of the performance means further suggested that participants tended to perform fastest when the workspace and head were aligned and progressively slower as the angular disparity between the two increased. However, only the right orientation resulted in progressively longer performance times as eyes-closed and head–workspace angular deviation increased from 0° to 45° to 90°. The slight symmetry observed in the central workspace condition between congruent and right workspace may reflect the predominance of right-handed participants, although this possibility remains speculative and should be examined in future studies using balanced samples of right- and left-handed individuals. At present, we can report that a disparity of at least 45° between direction of gaze in the absence of vision and workspace location negatively affects motor efficiency.

Several limitations should be acknowledged. First, the sample consisted primarily of right-handed participants performing a relatively simple construction task. Whether similar effects would be observed for more complex object manipulation or in left-handed individuals remains unknown. Second, the study measured task completion time but did not directly assess movement kinematics, head stability, or reaching trajectories. Motion capture or eye-tracking techniques could provide additional insight into how head orientation influences sensorimotor control throughout task execution. Finally, because the study was conducted with sighted participants who were temporarily deprived of vision, caution should be exercised when generalizing these findings to individuals who are congenitally or adventitiously blind. Long-term adaptations to blindness may alter the extent to which head orientation contributes to spatial representations and motor planning.

Further investigation that includes the examination of blind individuals is warranted. All participants were blindfolded, sighted adults. The sensorimotor adaptation patterns of congenitally or late-blind people differ greatly due to long-term cross-sensory plasticity, limiting the extent to which our findings may generalize to visually impaired individuals.

We view the present study as establishing an initial experimental framework for examining the contribution of head orientation to nonvisual fine motor performance rather than as providing evidence about motor behavior in blind populations specifically. Direct examination of congenitally, early-, and late-blind individuals represents an important next step. Finally, the use of more naturalistic tasks, such as those involved in activities of daily living (ADLS), may clarify the extent to which head orientation contributes to internal spatial representations independent of vision in an everyday context.

Despite these limitations, the present findings demonstrate that orienting the head toward a workspace facilitates manual performance even in the absence of visual information. These results extend previous research on perception–action coupling by suggesting that head orientation serves functions beyond directing visual attention. Instead, the findings support the view that head orientation is incorporated into an embodied sensorimotor representation that organizes action across multiple sensory modalities.

## Data Availability

The original contributions presented in this study are included in the article. Further inquiries can be directed to the corresponding author.
